# Artificial Intelligence Applications in the Process of Power Cable Operation and Maintenance: A Review

**DOI:** 10.3390/s26196192

**Published:** 2026-09-30

**Authors:** Shice Zhao, Qiansen Zhang, Zibo Yin, Hasiyeti Yesibolati, Yanjie Song, Zhaoyun Liu, Bin Wang

**Affiliations:** 1 Hebei Provincial Collaborative Innovation Center of Transportation Power Grid Intelligent Integration Technology and Equipment, Shijiazhuang Tiedao University, Shijiazhuang 050043, China; zhaoshice@stdu.edu.cn (S.Z.); 15188767822@163.com (Z.Y.); wangbin@stdu.edu.cn (B.W.); 2 School of Electrical and Electronic Engineering, Shijiazhuang Tiedao University, Shijiazhuang 050043, China; 3 Xinjiang Oilfield Company of PetroChina, Karamay 834000, China; 18309905308@163.com; 4 School of Information Science and Technology, Dalian Maritime University, Dalian 116026, China; songyj_2017@tju.edu.cn; 5 Shenxing Cable Group Co., Ltd., Shijiazhuang 052260, China; hedy@sxcables.com

**Keywords:** power cable operation and maintenance, artificial intelligence, condition monitoring, fault diagnosis, digital twin

## Abstract

Power cables are the core power transmission carriers for modern urban power supply, and their safe and reliable operation is crucial to the stability of urban power grids. Traditional cable operation and maintenance (O&M) mainly relies on manual experience, suffers from prominent data silos, and struggles to meet the demands of complex power grids and high-reliability power supply. Data-driven artificial intelligence has been increasingly applied in cable O&M, serving as a complement to conventional operation frameworks. We focus on the O&M process oriented towards fault handling. Firstly, attention is paid to the core physical quantities for cable condition monitoring and the current application status of Internet of Things (IoT) sensing technology. Subsequently, the limitations of traditional fault diagnosis methods are systematically analyzed. Furthermore, we highlight the research progress of deep learning models such as attention mechanisms and convolutional neural networks (CNNs) in cable fault diagnosis. Digital twin (DT) technology is also addressed, and its synergistic effect with AI technology in the intelligent O&M framework is systematically analyzed. Finally, we summarize the challenges faced in current technology implementation and look forward to future development directions.

## 1. Introduction

Power cables constitute a core component of modern urban power grids and clean energy transmission systems. Their operational reliability is directly related to the safe and stable operation of power systems [1]. Power cables have seen expanding applications and rising voltage levels driven by rapid urbanization and the ongoing energy transition in recent years. The power cables adopted in the current urban power transmission and distribution system are mostly medium-voltage cables above 10 kV. A large-scale and structurally complex cable transmission and distribution system has been established [2]. However, most cables are laid in complex and harsh environments such as underground tunnels and trenches. Meanwhile, a large number of cables are gradually entering their aging stage, leading to increasingly prominent degradation and failure of key components. Consequently, the difficulty of fault troubleshooting has risen substantially. Higher requirements have been put forward for the accuracy, timeliness and intelligence level of operation and maintenance technologies accordingly [3].

The limitations of traditional cable operation and maintenance modes have become increasingly prominent amid the severe operating conditions of cables, which are mainly reflected in the following aspects.

(1) Reliance on manual experience and lack of predictability. Operation and maintenance heavily rely on the subjective judgment and past practical experience of staff, making it difficult to reliably guarantee the accuracy and consistency of maintenance work. Moreover, most maintenance modes adopt passive emergency repair after faults occur, lacking the ability to predict potential risks. In the taxonomy of maintenance strategies reviewed by M. Moleda et al., such a passive mode is categorized as corrective maintenance, whose major drawback is the increased repair time and cost caused by unplanned downtime [4]. Accordingly, this mode may raise the risk of unplanned power outages and cause economic losses.

(2) Insufficient capability in data integration and utilization. Various types of information generated during the whole operation and maintenance process are isolated and scattered. Multi-source heterogeneous data can hardly be integrated efficiently. Y. Yan et al. pointed out that the data involved in grid digital operation, such as partial discharge signals and temperature, are generally multi-source and heterogeneous. This leads to low integration efficiency and slow anomaly detection response, making it difficult to support timely and reliable O&M decisions [5].

(3) Monitoring and maintenance modes are rigid. Most existing monitoring methods rely on traditional offline withstand voltage tests and manual inspections, making it difficult to achieve real-time and accurate perception of cable operating conditions. Maintenance cycles are mostly set regularly, such that they cannot be dynamically adjusted based on the actual health status of cables, causing an unbalanced allocation of operation and maintenance resources. The above problems have been further aggravated by the continuous expansion of the power grid and large-scale integration of distributed generation, making conventional approaches less adaptable to evolving grid conditions [6].

**Figure 1:**
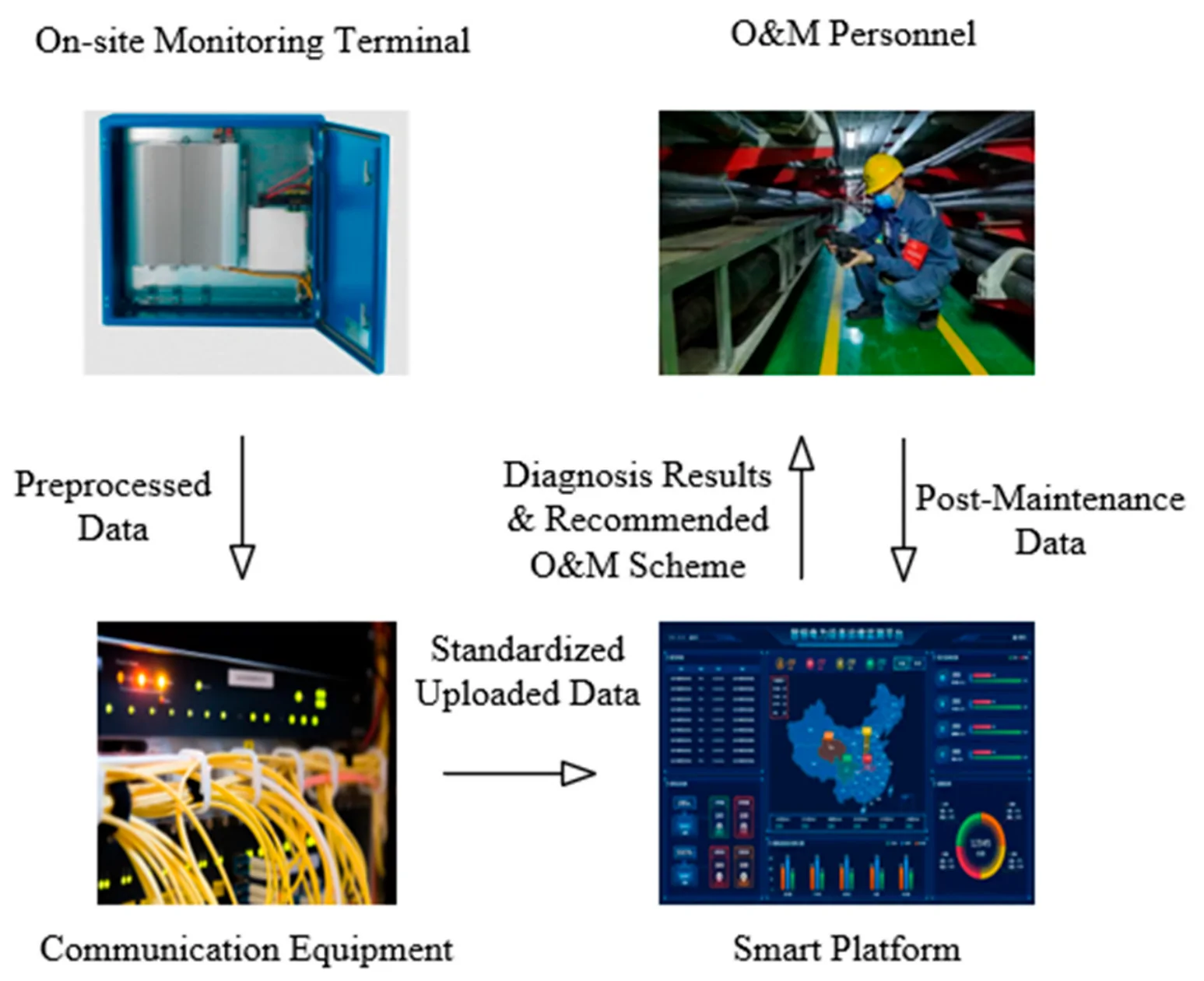
Block diagram of operation and maintenance process.

Artificial intelligence technologies have advanced in recent years, with improved capabilities in data processing, feature learning, and decision-making [7]. AI algorithms can extract valid features from large-scale multi-source datasets and deliver consistent fault analysis results when monitoring noise is low and sufficient labeled fault samples are available. AI models can attain high diagnostic accuracy for documented cable fault categories under standardized test conditions. H. Kumar et al. reviewed artificial intelligence-based classification methods for partial discharges in medium-voltage cables and reported recognition accuracies typically exceeding 90%, with CNN-based classifiers achieving up to 99.82%, attributing such performance to the automatic hierarchical feature extraction of deep models, which reduces the dependence on manual feature engineering [8]. AI technologies are enabling a gradual transition of cable O&M toward intelligent modes. Such tools can mitigate many inherent limits of traditional maintenance systems with complete sensing and data support, while practical deployment still has areas to be refined. In particular, recent research has expanded from traditional data-driven approaches to emerging paradigms such as physically constrained multimodal large language models, digital twin-enabled synchronous mapping, and multimodal signal collaborative diagnosis [9,10]. Although these paradigms have mainly been demonstrated in other equipment domains, they may appropriately motivate potential future research directions for cable fault diagnosis. We focus on the fault handling process in cable operation and maintenance, systematically exploring the key technologies of three core links: condition monitoring, fault diagnosis, and intelligent operation and maintenance decision-making, whose architecture is shown in Figure 1. As the core computing hub of the whole system, the smart platform receives standardized uploaded data for unified storage and management. It conducts intelligent fault diagnosis and digital twin-based scenario simulation. The platform further generates feasible recommended O&M schemes. These analysis results and suggested schemes are delivered to O&M personnel for decision reference. After the completion of on-site maintenance, post-maintenance data is fed back to the smart platform for permanent archiving, model iteration, and digital twin state updating.

Although artificial intelligence technologies have been increasingly applied across the entire process of power cable operation and maintenance, several recent reviews have addressed power cable monitoring and diagnosis from different perspectives. Y. Song et al. surveyed multi-parameter sensing and condition assessment technologies for power cables, covering both electrical and non-electrical parameters, but their work remained at the monitoring level without extending to diagnostic algorithms or intelligent maintenance decision-making [11]. S. Govindarajan et al. examined partial discharge diagnosis methods in cables, covering detection techniques, localization approaches, and the application of deep learning, yet their scope was confined to PD as a single monitoring modality [12]. S. Li et al. focused on defect inspection methods for composited cable terminals, involving terahertz imaging, ultrasonic testing, and digital twin technologies, but offered limited discussion of artificial intelligence methods [13]. Collectively, these reviews have provided valuable summaries of their respective sub-fields, advancing cable O&M research from the perspectives of sensing technologies, partial discharge diagnosis, and terminal inspection. Nevertheless, their scopes are confined to a single technical stage, a single monitoring modality, or a specific cable component, and have not yet systematically surveyed the application of AI technologies in condition monitoring, fault diagnosis, and intelligent operation and maintenance.

To address the above research deficiencies, the application of artificial intelligence in the three core operation and maintenance processes oriented towards cable fault handling is taken as the research focus. We first analyze the monitoring stage, reviewing the key physical quantities and their sensing devices, as well as the IoT perception technologies that collect and transmit the monitoring data. These monitoring data serve as important material for subsequent AI analysis. The fault diagnosis stage is then analyzed, focusing on the limitations of conventional methods. A systematic review is presented for three mainstream AI architectures, namely attention mechanisms, CNNs, and LSTMs, comparing their feature extraction capabilities, computational overhead, and deployment conditions. The intelligent O&M decision-making stage is finally explored, focusing on the collaboration mechanism between digital twin technology and AI. On this basis, we summarize the common challenges facing AI applications across the three processes and propose future directions. The remainder of this review is organized as follows. Section 2, Section 3 and Section 4 correspond to the three core processes, Section 5 discusses the existing challenges and future prospects, and Section 6 concludes the article.

## 2. Condition Monitoring for Common Defects of Power Cables

Cable condition monitoring technologies focus on defect characteristics and fault mechanisms throughout the full life cycle of power cables. This technology acquires relevant physical quantities such as cable insulation performance, operating conditions, and environmental parameters by means of multi-dimensional sensing. Data acquisition, transmission, and preprocessing are thereby completed [14]. Such collected data can support anomaly screening, cable health evaluation, and maintenance decision-making under adequate data matching conditions. Driven by the Internet of Things and intelligent sensing technologies, cable condition monitoring is gradually moving beyond simple discrete offline-sampling modes toward full-range online real-time perception.

### 2.1. Physical Quantities for Cable Monitoring

The physical quantities monitored in cables can be divided into electrical quantities and non-electrical quantities. Electrical quantities include key indicators such as partial discharge and sheath circulating current. Non-electrical quantities are mainly environmental and operating parameters such as temperature and humidity.

Partial discharge is a local breakdown phenomenon occurring inside the insulation of high-voltage cable systems under intense electric fields. Progressive long-term accumulation of such activity impairs insulation properties over time, making partial discharge an early precursor indicative of upcoming cable insulation failure [15]. The partial discharge magnitude of a cable reflects its insulation condition and the development of local defects.

The core principle of partial discharge monitoring is to capture and analyze multi-dimensional characteristic signals generated during the discharge process, including electrical, thermal, optical, and chemical signals [15]. Signal monitoring is mainly affected by factors such as sensor sensitivity, on-site noise, external electromagnetic interference, and signal attenuation. Therefore, one of the core challenges in partial discharge monitoring is to ensure that signals undergo no significant attenuation or distortion during transmission and truly reflect the actual discharge behavior of cable insulation. Another difficulty lies in signal analysis and extraction. It is necessary to separate the characteristic information that can accurately represent the cable insulation state from the detected mixed signals. Meanwhile, the influence of external interference and invalid signals should be eliminated. A comparison of various power cable partial discharge monitoring technologies is shown in Table 1.

For 10 kV and above power cables in distribution-network systems, high-frequency current transformer (HFCT), ultra-high-frequency (UHF), and acoustic emission (AE) are relatively widely adopted sensing approaches for on-line partial-discharge monitoring in practical engineering scenarios. Based on available studies and engineering experience, there is hardly a universal optimal sensor that can adapt to all operating conditions. Sensor selection usually needs to be comprehensively determined according to cable laying modes, grounding schemes, and monitoring locations. HFCT is generally applied to joint-related defect detection. However, under buried-cable operating conditions, it is susceptible to signal shunting effects and on-site electromagnetic interference, which may degrade its detection performance to a certain extent. UHF sensors generally deliver relatively high sensitivity at cable accessories, yet electromagnetic waves decay rapidly while propagating along cable main bodies. Acoustic-emission sensors can avoid interference originating from electrical electromagnetic noise. Nevertheless, restricted by acoustic attenuation characteristics, they are generally only applicable for short-range detection near cable joints. Given that single-sensor schemes mostly come with their respective inherent limitations, multi-sensor complementary configurations are frequently adopted in engineering practice to improve the reliability of defect detection.

Artificial intelligence mainly functions in the post-processing stage after signal acquisition. Its ability to compensate for various inherent hardware-level limitations of sensors remains limited under current technical conditions. To this end, robust partial discharge measurement often relies on the co-design of the sensing front end and signal processing. J. Chen et al. combined pulse-injection time synchronization with short-time Fourier transform and variational mode decomposition denoising. When the signal-to-noise ratio is as low as −11.33 dB and conventional envelope and wavelet methods have failed, the pulse arrival time can still be reliably extracted, and the method has been verified on laboratory 10 kV cables and field 110 kV cables [16]. AI methods can perform denoising, feature extraction, PRPD/PRPS pattern interpretation, and defect-pattern recognition for noisy raw signals output by sensors. They help mitigate the adverse interference brought by complex electromagnetic environments in distribution networks and reduce the probability of false and missing alarms to a certain extent. M. Zhu et al. showed that a one-dimensional deep learning denoiser can still recover PD pulses when the input signal-to-noise ratio is as low as −10 dB; in field tests on 110 kV cables, all 18 measured PD pulses were retained, and all 17 pulse-noise waveforms were removed [17]. It is worth noting that the relatively high recognition accuracy reported for most AI models is achieved based on well-controlled laboratory defect datasets. Constrained by practical factors such as the difficulty in obtaining sufficient on-site fault samples and domain-shift effects, model performance may degrade to varying degrees when transferred to practical operating conditions of 10 kV-and-above distribution networks.

The sheath circulating current can directly reflect the operational health of the grounding system and the sheath insulation. Its generation is directly related to the electromagnetic induction characteristics and grounding method of single-core cables. The passage of current through the conductor of a single-core high-voltage cable induces a voltage in the metal sheath. The cross-bonded grounding method is commonly used in engineering practice to suppress excessive induced voltage. However, due to factors such as unequal section lengths and unbalanced load currents, the induced voltage vectors in the sheath loop cannot be fully offset, thus forming a circulating current [18]. Excessive circulating current increases additional sheath losses, leading to elevated cable temperatures, reduced ampacity, and even thermal breakdown of the insulation.

Monitoring of the circulating current can provide early warning of common faults, including sheath open circuit, water ingress in cross-bonding boxes, and breakdown of joint epoxy prefabricated components. The circulating current is closely related to induced voltage, grounding resistance, and cable laying mode. At present, the commonly used monitoring method is online monitoring [19]. This method adopts power-frequency current sensors installed at the coaxial cable connections of cross-bonding boxes to realize real-time acquisition of sheath current data at each monitoring point. The collected data are compared with the values under normal and fault conditions. However, a key limitation is that measured sheath current includes both leakage and circulating components. This coupling effect can lead to false positives when leakage current fluctuates due to environmental moisture or surface contamination.

Temperature variation is closely related to cable insulation performance, operating conditions, and fault evolution. It directly affects the health status and operational risk of cables. XLPE cables have a clear rated operating temperature limit, with a maximum normal operating temperature of approximately 90 °C. The temperature of medium- and low-voltage cables can surge to 150 °C under overload and short-circuit conditions, while the insulation temperature of high-voltage cables can even reach 250 °C during short circuits [20]. Continuous excessive temperature accelerates the breakage of XLPE molecular chains and the decrease in crystallinity, leading to thermal aging of the insulation. It also exacerbates corrosion of the metal sheath and ablation of the water-blocking buffer layer. Such excessive temperature further degrades cable insulation resistance, raises the likelihood of partial discharge, and ultimately leads to catastrophic failures including insulation breakdown.

Real-time monitoring of cable temperature enables timely identification of fault precursors such as local temperature rise and abnormal hot spots induced by sheath circulating current and increased local loss. However, some insulation defects (e.g., early-stage partial discharge in voids) may not produce measurable temperature anomalies until they have progressed significantly, and early detection and treatment are conducive to preventing faults from further escalating. Long-term temperature monitoring data can reflect the aging trend of cable insulation. Combining temperature variation patterns with other monitoring information allows a more comprehensive assessment of the overall insulation condition of cables. High-voltage cable temperature monitoring currently employs distributed optical fiber temperature measurement systems, point fiber Bragg grating sensors, infrared thermal imagers, and wireless temperature sensors [21,22]. These technologies enable continuous temperature measurement along the cable route and non-contact temperature detection on the surfaces of joints and terminations.

### 2.2. Application of IoT Perception Technology in Cable Condition Monitoring

IoT sensing technologies constitute the perception layer of the power Internet of Things (IoT). They are responsible for collecting electrical parameters and environmental variables of cable systems and transmitting the data back for analysis [23]. IoT technology enables the collection and aggregation of cable-monitoring data, which can also serve as important material for artificial intelligence analysis. To fully tap the inherent information of monitoring data, intelligent algorithms are generally required for feature extraction and condition assessment. The actual effectiveness of their integration depends on data quality, sensor deployment density, and the communication transmission system connecting perception terminals and computing units.

IoT perception and transmission technologies adopted for cable condition monitoring can be divided into two categories, each with differentiated characteristics. The first category comprises low-power wide-area network (LPWAN) technologies represented by LoRa and Narrowband IoT (NB-IoT), which are used to access scattered independent sensors. The second category is distributed fiber optic sensing technology, represented by distributed acoustic sensing (DAS), which constructs a continuous sensing medium using the existing optical fibers along the cable.

LoRa operates in the license-free sub-gigahertz industrial, scientific, and medical (ISM) frequency band. In practical applications, the communication distance is affected by environmental factors: typically 2–5 km in urban areas and up to about 15 km in rural or open conditions, while the battery life of sensor nodes depends on the specific configuration and reporting duty cycle. However, its data transmission rate is only 0.3–50 kbps, which is insufficient for transmitting raw high-frequency partial discharge waveforms. Therefore, monitoring systems built on LoRa perform local preprocessing, extracting only characteristic indicators such as the number of partial discharge pulses and the maximum discharge amplitude, which are then compressed and uploaded. Tian Zhuang et al. developed a system based on this approach, constructing a cable wireless sensor network that transmits processed partial discharge feature parameters to a central monitoring unit via LoRa [24].

NB-IoT operates on licensed LTE frequency bands managed by operators and generally provides higher transmission rates and lower latency than LoRa, although its actual performance is constrained by operator network coverage and network conditions. This technology is more suitable for real-time alarm information transmission triggered by sudden faults such as abrupt partial discharge increases and sheath current anomalies. Its main limitation lies in its reliance on cellular network coverage, which often results in unstable LTE signals in cable tunnels and remote substation areas. Meanwhile, under battery-powered, periodic continuous reporting conditions, the average power consumption of NB-IoT modules is higher than that of LoRa, and continuous data reporting significantly reduces battery life [25]. In engineering practice, a hybrid architecture scheme has been adopted in several reported systems. Routine periodic low-power data uploads use the LoRa channel, while alarm-type high-priority information switches to NB-IoT as a backup transmission link. This scheme has been implemented in cable anti-damage monitoring systems and ultra-high-voltage cable digital twin platforms [26].

Distributed acoustic sensing uses optical fibers laid along the cable as a continuous sensing medium. Laser pulses are emitted into the fiber, generating Rayleigh backscattered light that carries acoustic and vibration disturbance information at every point along the fiber. With this characteristic, in specific systems, DAS has been reported to detect and locate various events over distances of up to 50 km with meter-level positioning accuracy [27]. Hao Wu et al. combined DAS with one-dimensional convolutional neural networks to extract vibration features along the cable, distinguishing external interference from internal cable faults, thereby reducing the false alarm rate in long-distance monitoring scenarios [28]. DAS technology faces two typical application bottlenecks in engineering deployment. First, continuous sampling along the entire optical fiber generates massive high-dimensional vibration data. If entirely reliant on cloud-based centralized processing, it can easily lead to excessive transmission bandwidth and storage resource consumption. Some studies have adopted edge computing units for front-end data preprocessing to alleviate this issue. Second, the vibration detection sensitivity of this sensing system is high, making it susceptible to interference signals from environmental disturbances such as road traffic and civil construction. Without supporting multi-dimensional noise reduction algorithms and intelligent event discrimination strategies, a prominent false alarm phenomenon is likely to occur [29].

Based on the performance characteristics of the three technologies reported in the reviewed literature, their differentiated monitoring scenarios can be summarized as follows. For low-frequency periodic data collection at discrete fixed measurement points such as cable joint temperature and cross-bonding box sheath current, LoRa offers good low-power suitability. Within cellular network coverage, sudden abnormal alarm information requiring reliable reporting, such as abrupt increases in partial discharge and sheath current exceeding limits, is more suitable for transmission via NB-IoT. For uninterrupted full-range monitoring of long-distance cable lines and identification of hazards without preset measurement points, such as external construction and ground deformation, DAS enables synchronous monitoring over several kilometers and provides meter-level positioning results. Combining DAS for full-range distributed sensing, LoRa for fixed-point periodic data collection, and NB-IoT for alarm uplink transmission can adapt to various cable laying environments, including tunnel laying, direct-buried suburban lines, and substation outgoing lines, thereby improving line monitoring coverage.

From the perspective of current technology evolution trends, the combined application of IoT sensing technologies and artificial intelligence algorithms is gradually advancing in the field of cable operation and maintenance monitoring. Due to the inherent bandwidth limitations of LoRa and NB-IoT communication, monitoring systems built on LPWAN tend to deploy lightweight AI inference modules at edge terminals to complete local data feature extraction and preliminary screening. For the large-scale time-series sensing data generated by DAS systems, existing research often embeds deep learning models within demodulation equipment to achieve real-time vibration interference identification and noise reduction, thereby reducing the communication overhead caused by invalid data transmission. The transmission bandwidth, communication latency, and spatial coverage of IoT terminals constrain, to a certain extent, the deployment location and computational architecture of intelligent algorithms.

Against this background, the hierarchical distributed intelligent architecture is gradually becoming a widely discussed technical route in the cable monitoring field. Based on existing research, this direction represents an emerging research hotspot in academia: although several small-scale pilot applications have appeared, large-scale deployment across the cable O&M industry has not yet been realized. Within this architecture, front-end sensors perform basic data preprocessing; gateway nodes aggregate monitoring data from multiple measuring points and implement intermediate-level information fusion; cloud platforms undertake computationally intensive fault diagnosis and condition assessment. Such an architectural pattern is expected to provide references for the subsequent design of intelligent cable monitoring systems.

## 3. Diagnosis Method for Cable Faults Based on Real-Time Monitoring Data

Cable faults are caused by the long-term coupling of multiple factors, including electrical, thermal, mechanical, and environmental stresses. Insulation degradation, structural damage, and external environmental impacts are the three main categories of triggers. Permanent failures will gradually form if potential early faults cannot be identified and handled in a timely manner, thereby causing power outages and threatening the safe and stable operation of power grids.

For a long time, cable fault diagnosis has mainly relied on traditional signal analysis and electrical measurement technologies. These methods have been widely used in cable operation and maintenance due to their clear principles and practical engineering applicability. However, power grid structures are growing increasingly complex, and distributed generation is being extensively integrated. As a result, traditional techniques may not be fully adequate for complicated operating scenarios. This has driven fault diagnosis methods to evolve toward data-driven artificial intelligence [30].

### 3.1. Conventional Approaches for Cable Fault Diagnosis

Cable fault diagnosis centers on fault detection and fault location. Fault detection identifies fault occurrence and classifies fault types by analyzing electrical parameters and signal characteristic variations during cable operation, and acts as a prerequisite for fault location. Conventional fault detection methods mainly analyze variations in electrical parameters in the time or frequency domain. These parameters include traveling waves, grounding current, and zero-sequence current. Diagnostic conclusions are then drawn through corresponding calculation and comparison [31]. Commonly used methods at present include traveling wave detection and grounding current analysis. Some improved schemes integrate power frequency information with traveling wave data, thus enhancing the ability to evaluate the health status of distribution network feeders [32]. In 2022, N. Peng et al. proposed a measurement method based on the grounding current at cable terminals. This method adopts techniques such as the modal signal decoupling algorithm to realize rapid detection of short-circuit and arc faults [33].

Fault location is a technique that accurately determines the spatial location of faults based on fault detection via signal analysis and mathematical calculation. Its core objective is to provide guidance for intelligent operation and maintenance and shorten fault repair time. Conventional fault location is centered on traveling wave ranging technology, which can be divided into single-ended and double-ended detection modes. The single-ended traveling wave method analyzes the arrival time and waveform characteristics of fault traveling waves. It reduces the sampling frequency requirement and improves positioning sensitivity by combining optimization algorithms such as three-dimensional subspace analysis and angle calculation of fitted line segments [34]. The double-ended traveling wave method relies on discrete wavelet transform, pole-symmetric mode decomposition, and other signal processing techniques. It enhances adaptability to wave speed variations and line parameter uncertainty [35,36]. In addition, frequency-domain analysis methods establish a mapping relationship between frequency-domain response and fault location through techniques such as the fast Fourier transform and numerical Laplace transform. Time-domain analysis methods estimate fault locations by establishing time-domain fault models, transforming the positioning problem into linear equations, and performing numerical solutions.

Traditional fault diagnosis methods have achieved relatively widespread practical application in engineering operation and maintenance scenarios. However, this category of techniques still has several inherent limitations. These methods typically require the construction of mathematical models with high complexity. Fault feature extraction demands substantial professional experience from practitioners, and relevant parameters need manual pre-tuning. In complex operating environments with multiple interferences, they are susceptible to noise signal interference, and the accuracy of fault detection and localization tends to decline. Meanwhile, the transferability of algorithms across different field operating conditions is limited, leaving room for optimization in overall generality and operational stability. In light of these shortcomings, existing research is progressively shifting toward data-driven diagnostic approaches, relying on field-measured data to autonomously learn fault discrimination logic and reducing the dependence of diagnostic algorithms on prior analytical physical models.

### 3.2. Application of Artificial Intelligence Technology in Cable Fault Diagnosis

**Figure 2:**
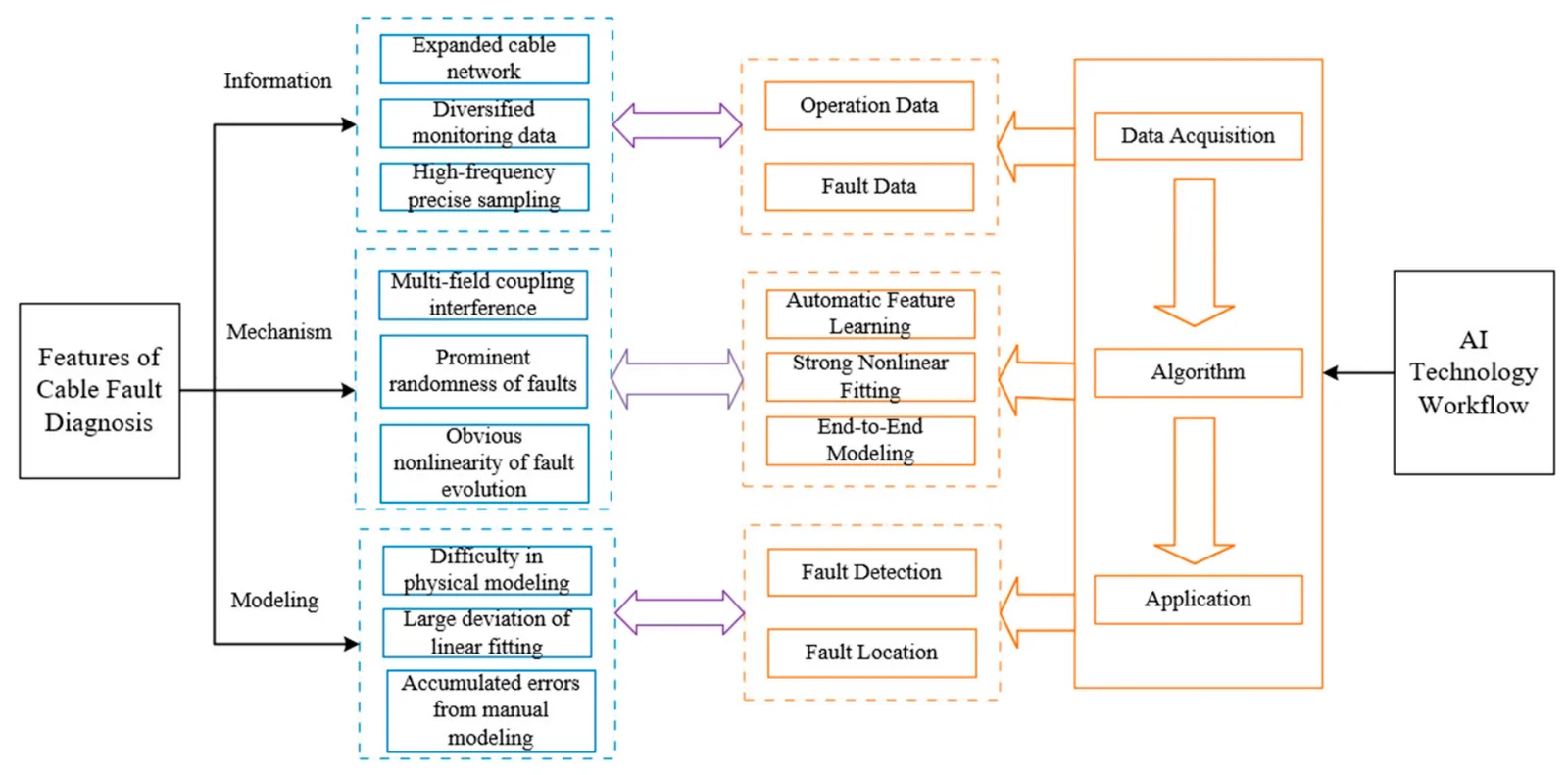
Framework for mapping cable fault diagnosis features to artificial intelligence technical workflows.

The volume of monitoring data for power-system operation and maintenance has grown substantially, driven by the digital construction of smart grids. Traditional cable fault diagnosis is constrained by three factors: data scale, fault mechanisms, and modeling difficulty. Machine learning and deep learning, as common implementation forms of artificial intelligence, operate through the technical chain of data acquisition, algorithm analysis, and engineering application, performing autonomous feature learning, nonlinear fitting, and end-to-end modeling for fault detection and localization. Such data-driven diagnostic methods have been extensively studied and applied in the field of cable fault diagnosis [37]. Figure 2 presents the correspondence between typical features of cable fault diagnosis and the artificial intelligence technical workflow. It summarizes cable fault diagnosis characteristics from information, mechanism, and modeling perspectives and depicts their mapping to the AI technical workflow.

Machine learning-based fault diagnosis approaches can adaptively learn decision boundaries from data, reducing reliance on manually preset thresholds that are required in traditional methods. They are capable of handling diverse and complex fault signals, thus improving the accuracy and efficiency of fault detection and location [38]. A. Ansari et al. conducted a comparative study on medium-voltage XLPE-insulated underground cables. They pointed out that conventional threshold-based approaches miss 20–30% of early degradation signs. In contrast, machine learning-based methods achieved significantly higher detection accuracy, with approximately 12% improvement over traditional approaches. The same study also demonstrated a 30% reduction in unplanned failures and a 25% decrease in maintenance costs through proactive ML-based maintenance [39]. However, such methods rely on feature engineering and statistical learning algorithms for data modeling and prediction, making them difficult to apply to more complex modeling scenarios, which limits their application to more complex modeling problems.

Traditional machine learning fault diagnosis schemes typically combine manual feature engineering with statistical learning algorithms to construct prediction models. Such architectures have limited adaptability to high-complexity fault modeling scenarios, and their scope of application is correspondingly constrained. Against this background, deep learning-based cable fault diagnosis technology has gradually gained research attention and practical application. Deep learning falls within the domain of machine learning, adopting multi-layer neural networks as its foundational architecture, where network layers progressively parse sample data and generate hierarchical feature representations. The output effectiveness of the model fluctuates with sample size, environmental noise, and other operating conditions, while sufficient high-quality datasets can fully leverage the technical advantage of automatic multi-layer feature extraction inherent in this architecture [40]. For high-dimensional, nonlinear, noise-contaminated data, deep learning offers a data analysis logic distinct from traditional machine learning, reducing the involvement of manual feature design. Numerous studies have taken this as a starting point to conduct cable fault feature extraction and diagnostic analysis under complex power grid operating conditions.

#### 3.2.1. Attention Mechanism

**Figure 3:**
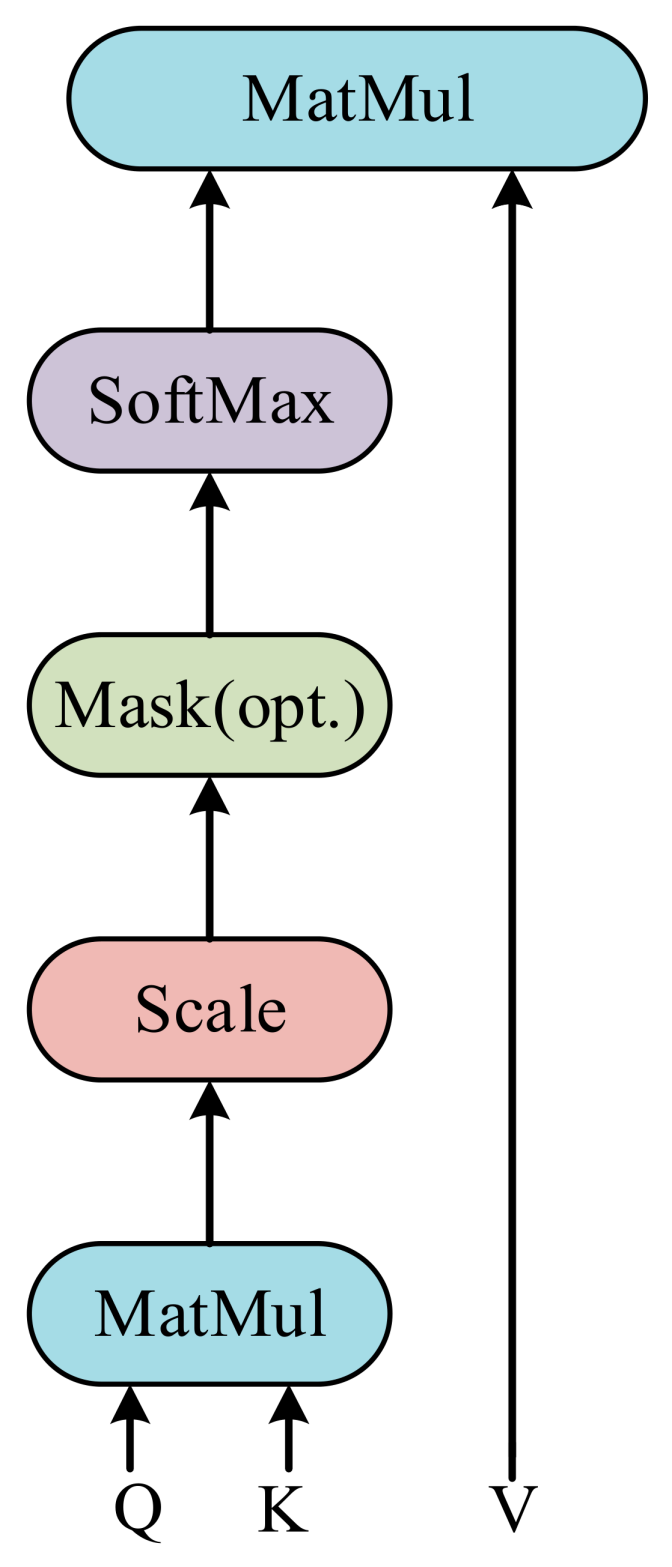
Flowchart of the attention mechanism.

Attention refers to the ability of the human brain to intentionally or unintentionally select a small portion of useful information from massive inputs for focused processing while ignoring other information. Experts and scholars proposed the attention mechanism based on studies of human visual cognition. Its essence is to achieve efficient allocation of information processing resources [41]. The attention mechanism focuses on important information with high weights, ignores irrelevant information with low weights, and can adjust the weights dynamically. Thus, it can select key information under different conditions, thereby achieving higher scalability and robustness. Mathematically, the input sequence and target sequence are converted into three sets of vectors. They are query vectors, key vectors, and value vectors, denoted as *Q*, *K*, and *V*, respectively. Weights are generated by calculating the similarity between query vectors and key vectors, and the output is obtained through the weighted aggregation of value vectors. Figure 3 shows the schematic diagram of this process, and the output matrix is obtained by Equation (1).


(1) $$Attention(A,K,V)=\mathrm{softmax}(\frac{QK}{\sqrt{d_{K}}})V$$

In the formula, softmax is a common activation function, and *d_k_* denotes the dimension of *K*.

Monitoring signals collected from cable sites are generally contaminated with environmental noise and redundant components. This can easily mask effective fault features and increase the difficulty of fault identification and localization. The attention mechanism can assign higher weights to key diagnostic components in the signal while attenuating irrelevant interference. Relevant schemes have been applied to cable fault detection, pattern recognition, and fault localization. Existing studies have adopted different attention embedding structures for model improvement to address various technical challenges in the feature extraction stage.

C. Pan et al. combined self-attention with LSTM to construct an SA-LSTM model for underground cable burial depth identification [42]. This approach addressed the challenge of fully capturing global dependencies in time-series transient electromagnetic signals. The model relies on self-attention to complete temporal correlation modeling and adaptive feature weight allocation. L. Yang et al. embedded SE channel attention into the YOLOv5 detection network [43]. This addressed another feature extraction difficulty, where weak fault features in the image channel dimension are easily obscured by complex backgrounds. Adaptive channel screening was used to highlight subtle fault pixels. Comparative experiments were conducted on a self-constructed drone-captured cable image dataset. Compared with the original YOLOv5, the model precision improved by 1.8% and recall by 2.1%, showing improvement in weak defect detection metrics.

Compared with other single-architecture networks, Transformer architectures directly associate arbitrary positions within a sequence, giving them an advantage in capturing global correlation patterns. Accordingly, they are suited to cable fault and partial discharge signals with long temporal spans and high feature coupling, and have been applied to cable fault pattern recognition tasks. Y. Li et al. introduced Swin Transformer into cable partial discharge recognition scenarios [44]. Window self-attention and shifted-window mechanisms were adopted to mine long-range global features, and an adaptive learning rate strategy was incorporated to optimize the model training process. Experimental results showed that the method achieved a recognition accuracy of 97.52%, with a maximum performance improvement of 6.89% over the baseline model.

However, pure Transformer structures have limitations in extracting shallow local details and subtle fault features from signals, and it is difficult to balance global correlation modeling and local feature representation. For this reason, the academic community has generally adopted a multi-network fusion improvement approach. The CNN–Transformer hybrid architecture has been increasingly adopted in recent studies, which combines the local feature extraction advantages of convolutional networks with the global-modeling advantages of Transformers. N. Zhao et al. adopted convolution operations at the front end to extract shallow detail features from signals [45]. Transformer multi-head attention was then used to capture instantaneous local features and periodic global features. In their experiments, the hybrid model achieved accurate classification of the four insulation-defect types on the constructed dataset.

Combined with the above attention-based schemes for cable diagnosis, different architectures exhibit diverse trade-offs in modeling capability, computational overhead, and data preprocessing cost. Lightweight embedding of SE channel attention into detection networks brings marginal increases in inference load, which is suitable for low-power UAV airborne inspection devices. This module mainly completes feature recalibration in the channel dimension. It lacks the ability to construct cross-region global image associations, which may increase the risk of undetected micro defects in complex working conditions. Swin window attention reduces computational pressure in global modeling through block-based partitioning and has been applied to small-sample partial discharge recognition tasks. When applied to two-dimensional spectral recognition, this approach requires converting time-series data into feature maps. Window segmentation may destroy the integral structural features of large-scale discharge signals under inappropriate window sizes. CNN combined with multi-head attention jointly models local waveform details and global dependencies. However, multi-layer stacked structures usually induce considerable computational consumption. Such unoptimized models are more applicable to offline experimental analysis, while facing deployment challenges in portable inspection terminals. In general, most lightweight attention structures improve deployment feasibility at the cost of partial global modeling performance. Compared with lightweight channel attention, global attention schemes usually achieve higher accuracy in complex recognition tasks, but require higher hardware and computational support, resulting in relatively higher deployment thresholds. Therefore, suitable attention schemes should be selected according to actual signal characteristics and terminal computing conditions in practical engineering scenarios.

Overall, research on attention-based models for cable fault diagnosis keeps advancing. Early channel and spatial-attention modules mostly served as auxiliary components to screen and enhance local fault features. Since then, lightweight global self-attention schemes represented by the Swin Transformer have been increasingly studied. Such models generally convert time-series signals into two-dimensional maps as input, and can mine global correlation features with computational costs kept under control. At present, numerous studies adopt attention-based hybrid architectures to consider both local waveform details and long-term fault evolution characteristics. Nevertheless, multi-head self-attention brings high computational overhead when processing long-sequence monitoring data, and its direct deployment on compact on-site inspection terminals will be affected by factors such as sequence length, model size, and terminal hardware capabilities. To address this issue, lightweight attention modules and CNN-Transformer hybrid frameworks are two key technical solutions being investigated in this field.

#### 3.2.2. Convolutional Neural Networks

**Figure 4:**
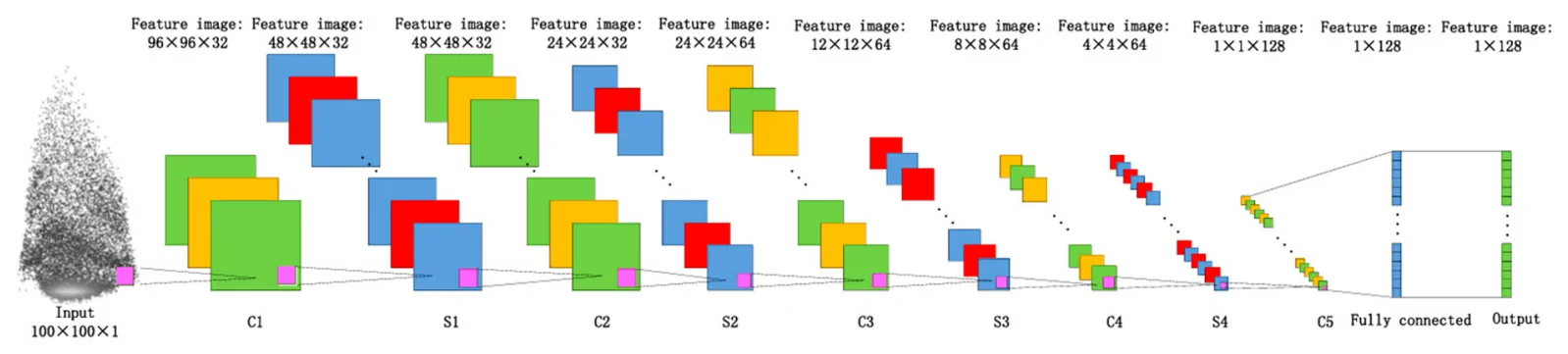
Structure of CNN algorithm.

Convolutional Neural Networks (CNNs) are classic deep learning models inspired by the biological visual cortex. Typically composed of alternating convolutional, pooling, and fully connected layers, a CNN can automatically learn hierarchical features from raw data in an end-to-end manner. It is widely used for processing high-dimensional data such as images and time series [46]. Figure 4 illustrates a typical CNN architecture.

CNNs are designed based on local receptive fields and weight sharing. The number of parameters and model complexity are relatively low. Pooling operations endow the network with a certain degree of translation, deformation, and scale invariance. The impact of input perturbations on feature extraction results is relatively small. Such networks can automatically learn hierarchical and progressive abstract features from raw data. They do not rely on manually designed features, thereby reducing the possibility of introducing subjective bias in the feature engineering stage. Meanwhile, the CNN architecture is scalable. Increasing network depth under controllable gradient propagation can enrich the dimensions and granularity of feature representation. With sufficient training samples, CNNs demonstrate good fitting and adaptation capabilities for high-dimensional, nonlinear, noise-contaminated data.

In cable fault diagnosis, fault features such as partial discharge pulses, traveling wave fronts, and current distortions are mostly local abrupt signals that possess translation invariance. This characteristic fits well with the local feature extraction logic of CNNs. Existing studies have explored various fault scenarios and technical paths using these networks. For identifying cross-bonding misconnection faults in sheath systems, J. Tu et al. employed CNNs to mine deep features from current phasor differences. Their model classified 36 wiring states and achieved a detection accuracy of 98.56% on their experimental dataset [47]. To address the confusion between early faults and disturbance signals, M. H. Wang combined wavelet transform with CNNs to construct a classification model. By optimizing the training strategy, overfitting was alleviated, and early-fault identification was improved in the corresponding test scenarios [48]. These studies respectively focus on expanding fault scenarios and integrating signal preprocessing. The results of these studies under their respective experimental settings reflect CNN’s technical strengths in local feature mining and multi-condition classification tasks.

One-dimensional convolutional neural networks (1D-CNNs) are CNN variants specially designed for one-dimensional sequence data. Different from two-dimensional CNNs operating on grid-based image inputs, 1D-CNNs perform convolution directly along data vectors, followed by max-pooling and fully-connected layers to extract local sequential features. Owing to this structural property, 1D-CNNs can accomplish feature learning, pattern classification, and regression tasks with notable computational efficiency, which makes them available for analyzing transient time-series signals collected from power cables. A. Said et al. proposed a hybrid diagnosis framework integrating a binary support vector machine (BSVM) and a one-dimensional CNN. For single-terminal transient-current signals, fractional discrete cosine transform (FrDCT) combined with singular value decomposition (SVD) was used for time–frequency feature extraction and dimension reduction. BSVM performed preliminary fault detection, and one-dimensional CNN further realized fault-type identification and regression-based fault-distance calculation. Under simulated conditions, this method achieved a classification accuracy of 99.6%, a maximum fault-location error of 0.095%, and an inference time of only 0.15 s [49]. However, this approach requires manually-designed time–frequency preprocessing, and its performance is only validated with ATP-EMTP simulation datasets, which has not yet been further substantiated by field-measured data.

In cable fault-localization research, some studies aim to balance localization performance and hardware cost by cutting down sensor quantities and relaxing sensor-performance demands for fault diagnosis. Sheath-current signals carry faint traveling-wave rising edges, which are hard to identify directly. T. Lan et al. adopted the sum of three-phase sheath currents as input and employed a CNN to detect the arrival of coaxial-mode traveling-wave wavefronts. In their scheme, sheath-current time-series waveforms are first sliced. The obtained two-dimensional time–frequency features are fed into the CNN after wavelet decomposition extracts detailed coefficients and min-max normalization is performed. Convolution-pooling layers extract latent waveform features for wavefront-arrival recognition. The mode value is taken from multiple sliced outputs to suppress recognition errors. Fault distance is finally computed based on the wavefront time difference between two cable terminals. Under 30 dB noise, simulation results on a 110 kV cross-bonded cable showed an average localization accuracy of 99.39% on 24 unseen fault cases. The accuracy remained largely unchanged when the fault resistance was raised to 500 Ω and when the cable formation was changed to a trefoil layout, degrading to 96.99% only when the noise level dropped to 10 dB. These results indicate moderate robustness against variable fault resistances and diverse cable structures. Nevertheless, its performance degrades under heavy noise. Since all validations are carried out on the PSCAD/EMTDC simulation platform, further support from field-measured data is still required [50].

**Figure 5:**
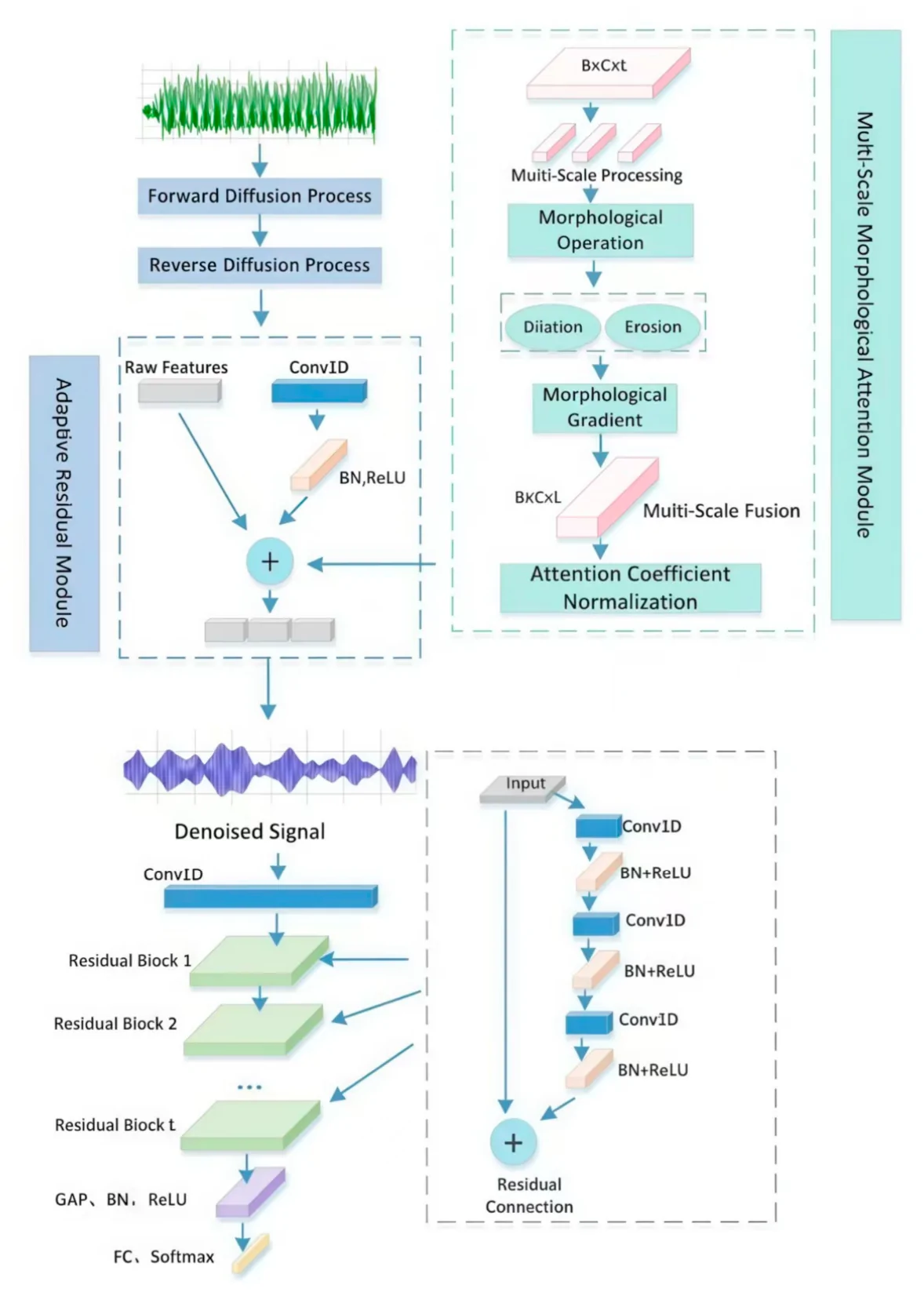
Network architecture of ARDDMA-Net [51].

Deep CNNs tend to suffer from gradient degradation as the number of layers increases. Residual connections are a typical structural improvement to mitigate this issue. Recent studies have further combined residual structures with denoising and attention mechanisms to optimize model performance under strong noise. L. Chen et al. proposed the ARDDMA-Net architecture (Figure 5), which integrates an adaptive residual diffusion denoising unit and a morphological attention module. The diffusion denoising unit suppresses background noise while preserving partial discharge pulse characteristics, and the morphological attention module enhances key features such as pulse width and edge structures. A multi-scale attention mechanism is then used to optimize the classification performance of the one-dimensional residual network. Under experimental settings with low signal-to-noise ratio and missing data, this study observed improvements in both recognition accuracy and robustness for partial discharge identification [51]. Such multi-module fused residual network designs complement the feature extraction capability of deep CNNs in complex noise and incomplete data scenarios, representing a technical exploration path for adapting such models to complex operating conditions.

We summarize the relevant characteristics of various CNN models mentioned above to compare the application differences in different convolutional architectures in cable fault diagnosis tasks. The comparison results are shown in Table 2. The accuracies reported in the table are obtained from different tasks, datasets, sample sizes, and validation conditions, and should not be interpreted as direct performance comparisons between models; nevertheless, the table can still, to some extent, reflect differences in structural and design trade-offs among different models. It can be observed from the comparison in the table that CNN-based models for cable fault diagnosis exhibit certain performance trade-offs. Improved convolutional networks designed for higher recognition accuracy and anti-interference capability mostly rely on large-scale simulation or laboratory datasets, limiting their practical applicability under certain working conditions. Lightweight convolutional schemes can reduce training data requirements, yet they generally show limited feature adaptability under diverse and complex fault conditions. From the perspective of existing research progress, early CNN-based cable fault diagnosis mainly adopted two-dimensional convolutional schemes to process PRPD patterns and time–frequency features. With the demand for end-to-end time-series modeling, lightweight one-dimensional time-series convolutional networks have been used in relevant studies. On this basis, improved deep CNNs integrated with residual structures have been adopted in many existing studies. CNNs with different structures can be applied to sub-tasks such as sheath topology identification, partial discharge detection, and traveling-wave location.

Nevertheless, convolutional architectures suffer from inherent structural limitations. Their feature extraction mechanism naturally focuses on local waveform information, resulting in weak capability in capturing global correlations within long time-series fault data. High-precision convolutional models usually entail high deployment requirements, while lightweight models present restricted applicability in complex fault scenarios. Most training schemes rely on simulation datasets, which may lead to degraded generalization performance under cross-condition scenarios. Constrained by the intrinsic drawbacks of pure convolutional structures, a single CNN framework can hardly simultaneously satisfy the requirements of global modeling capability, low computational overhead, and cross-scenario generalization. Under such circumstances, Transformer architectures with global time-series modeling capability appear in cable fault diagnosis research. At present, several technical directions are investigated in cable fault diagnosis. The fusion design of CNN and self-attention mechanisms is adopted to compensate for insufficient global-feature extraction by convolution. Further optimization of lightweight residual CNNs is carried out to accommodate practical deployment conditions. Small-sample transfer learning strategies are developed to reduce the demand for large-scale annotated fault samples.

#### 3.2.3. Long Short-Term Memory Network

**Figure 6:**
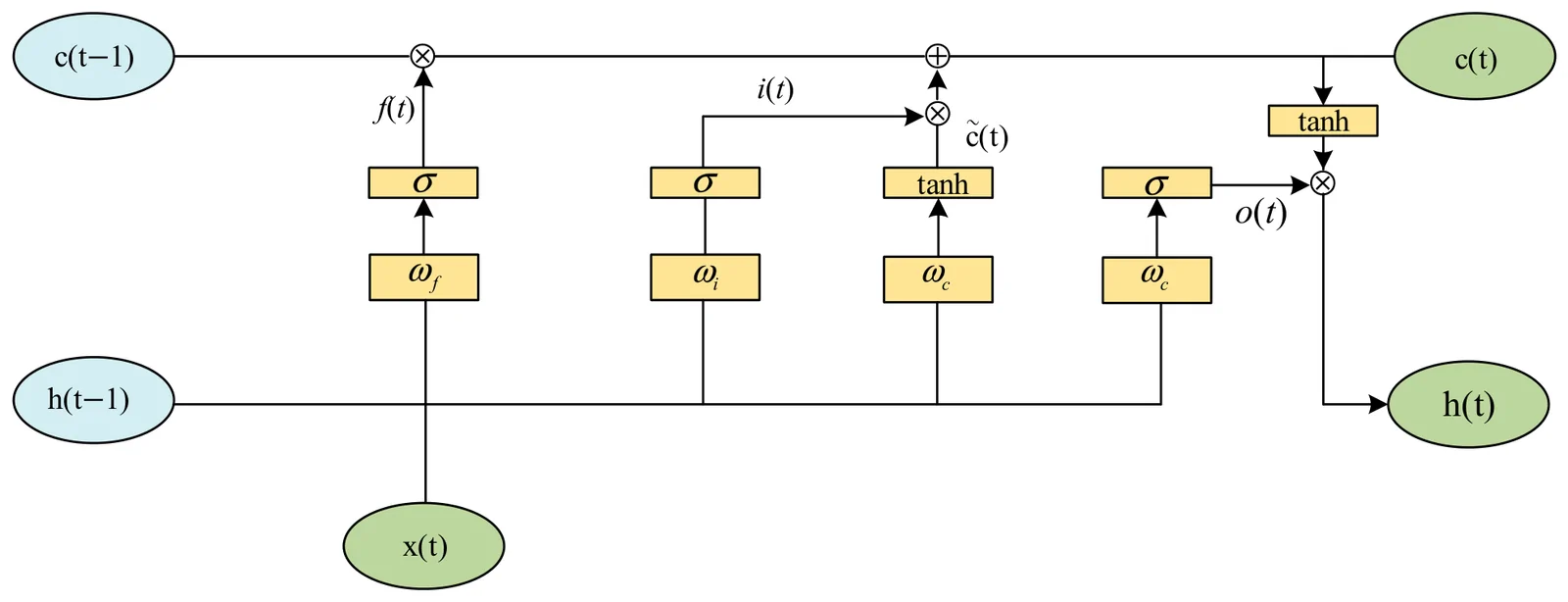
Schematic diagram of LSTM.

Long Short-Term Memory (LSTM) is an improved recurrent network proposed to address gradient vanishing and gradient explosion issues of traditional RNNs in long-sequence processing. LSTM adopts memory units and a gated structure including input, forget, and output gates to realize selective memorization, forgetting, and updating of time-series information. This network can capture long-term dependencies in sequential data. The working mechanism of LSTM is illustrated in Figure 6. The memory cell acts as the core component, and three types of gates cooperate to regulate information flow. The forget gate filters redundant historical information from the cell state. The input gate processes new information at each time step and updates the cell state. The output gate determines the current output based on the cell state and hidden layer features. The specific operation process is described as follows. The input sequence is first filtered by the forget gate to eliminate irrelevant historical data. The input gate generates updated values through the Sigmoid and Tanh functions. These updated values are multiplied with the original cell state element-wise to complete state iteration. Ultimately, the output gate combines cell state and hidden layer activation values to produce the output at the current time step.

Long Short-Term Memory (LSTM) networks alleviate the temporal gradient vanishing problem of traditional RNNs. This model adapts to the temporal evolution characteristics of cable insulation degradation and is suitable for time-series signal processing scenarios, including partial discharge detection and aging monitoring. T. Zhou et al. adopted a unidirectional LSTM to recognize multiple insulation defects of XLPE cables. Taking temporal harmonic features as inputs, the model detects typical defects including voids, protrusions, and water trees, and achieves a recognition accuracy of 95.83% on simulation datasets [52]. Unidirectional LSTM only analyzes time-series data in a single direction due to its inherent structural constraints, and its serial computation dependency limits parallel acceleration capability. The model suffers from gradual decay of early weak fault features in long-sequence cable monitoring, which leaves room for improvement in identifying early anomalies at complex structural positions such as cable joints. As verified by S. J. Chang et al., unidirectional temporal models fail to fully exploit bidirectional contextual information. The models cannot effectively distinguish inherent impedance reflection signals from actual defect signals at cable joints under certain operating conditions [53].

Bi-LSTM has been gradually applied to cable fault recognition to optimize temporal information utilization. It extracts contextual correlation features through bidirectional temporal paths and improves the overall temporal representation capability. S. S. Roy et al. adopted a Bi-LSTM model with Bayesian hyperparameter optimization for five types of XLPE cable partial discharge defect identification. This model reaches an accuracy of 97.33% on laboratory sample datasets [54]. In addition to bidirectional structural improvement, the hybrid architecture combining convolutional and temporal networks has also been widely adopted. Such architectures extract local waveform features via CNN and mine temporal evolution rules via LSTM, making up for the limitation of single temporal models and realizing the joint representation of spatial and temporal multi-dimensional features. R. Swaminathan et al. developed a CNN-LSTM hybrid architecture based on this fusion paradigm. It fully integrates the dual feature extraction advantages of CNN and LSTM for underground cable fault analysis, enabling simultaneous fault classification and localization. The model achieves a fault classification accuracy of 99.60% and an average localization error of 24.1 m [55].

The above two architectures are widely adopted in cable fault diagnosis owing to their capability in modeling the temporal dependencies of fault signals. These models still suffer from adaptation limitations in practical engineering deployment, stemming from the inherent constraints of basic recurrent structures. Their feature extraction process is susceptible to environmental noise, resulting in limited performance in screening short-duration core temporal segments such as discharge pulses and fault transients. Meanwhile, these models impose high inference costs when processing long-sequence samples, which hinders their lightweight deployment on edge terminals. Attention modules are integrated with temporal networks in existing studies to compensate for insufficient feature screening capability. Adaptive weight allocation is adopted to optimize the feature contribution of each time step, thereby suppressing invalid temporal interference and improving the model’s ability to focus on core fault signals. Nevertheless, the embedding of attention modules introduces additional computational overhead, which further increases the computing pressure of recurrent networks. To meet the requirements of edge deployment, lightweight temporal networks have been developed through parameter compression, pruning, and quantization to balance model recognition accuracy and real-time inference performance. Such lightweight operations impair the fine-grained feature extraction capacity of networks, which is an inherent limitation of general model compression techniques. These defects may result in unstable recognition of low-amplitude degradation signals, including water tree defects and incipient partial discharge.

#### 3.2.4. Comparative Analysis and Emerging Directions

The aforementioned architectures have been widely applied in cable fault diagnosis, yet each model may be constrained by its own structural characteristics and applicable conditions in practical engineering scenarios. LSTM, through its gated mechanisms and memory cells, is advantageous in long-range temporal dependency modeling, and has been mainly applied to diagnostic tasks centered on the temporal evolution of fault signals, such as analyzing the evolution patterns of fault waveforms and sheath currents, though its sequential computation limits parallel efficiency. CNN, with its local receptive fields and weight-sharing mechanism, has been widely applied to tasks dominated by local and transient features, such as PRPD pattern recognition and early fault warning, and lightweight one-dimensional CNNs further support deployment on front-end terminals; however, the receptive field of CNN is constrained by the kernel size, making it difficult to directly capture long-range dependencies among global features. In contrast, the Transformer, through the self-attention mechanism, directly associates arbitrary positions in a sequence and achieves stronger capability in grasping global features, and is generally advantageous in tasks dominated by global correlation modeling, such as complex fault pattern recognition, though its high computational overhead and strict dataset scale requirements create practical constraints on long-sequence processing and deployment on resource-limited edge terminals. From a maturity perspective, most of the reviewed LSTM-, CNN-, and Transformer-based studies have been validated on simulation or laboratory datasets, whereas field-measured validation remains limited across all three architectures. Accordingly, evaluation of their actual performance should take into account their respective experimental conditions.

Table 3 provides a comparative analysis of mainstream deep learning models and selected hybrid models from four aspects: computational cost, applicable fault types, data requirements, and real-time processing capability.

Comparison shows that the models differ in their respective strengths, but they all generally exhibit a high demand for high-quality data. To reduce this dependence, early methods mostly employed lightweight image transformations and interpolation-based oversampling such as SMOTE. SMOTE generates synthetic samples by interpolating between adjacent minority-class samples. Such methods are simple to implement and can balance class distributions to some extent; however, the generated samples remain close to existing ones, and the newly introduced feature variation is relatively limited [56]. Generative adversarial networks, by contrast, learn the distribution of real samples and generate new data accordingly. In recent years, such methods have been continuously improved, and their application in data augmentation for cable fault diagnosis has been deepened. K. Liu et al. combined TimeGAN-based time-series augmentation with few-shot learning, achieving a recognition accuracy of 90.73% in partial discharge diagnosis of high-speed train cable terminals using only 10% labeled data [57].

However, data augmentation only increases the number of samples and does not change the nature that models learn statistical correlations from data alone; under small-sample or cross-condition scenarios, generalization may still degrade, and even results contrary to physical laws may be produced. The generation and propagation of cable faults are governed by well-established mechanisms such as insulation degradation and traveling wave propagation; introducing these physical laws as prior knowledge into neural networks and constraining the model’s solution space provides a route to learning mechanism-consistent features under small-sample conditions. In the field of rotating machinery, studies have embedded fault dynamics equations as constraints or regularization terms into training to guide the network toward mechanism-consistent features: networks guided by physical constraints can still discriminate fault features under zero-fault-sample conditions [58], while models incorporating physics-consistency loss can reduce complexity and improve robustness while maintaining accuracy [59]. This line of thinking has recently begun to migrate to cable scenarios. X. Su embedded telegrapher-equation residuals and boundary consistency constraints into a physics-informed neural network, combining LSTM with a graph convolutional network to achieve medium-voltage cable fault detection and localization; in PSCAD/EMTDC simulations, even when the training set is reduced to 25%, its detection F1-score can still reach 94.1% [60]. A. Aguila Téllez et al. introduced physics regularization into a hybrid learning framework for fault location and classification of aged underground cables under parameter drift conditions [61]. Nevertheless, the above physics-fusion studies in the cable domain are still mainly validated through numerical or electromagnetic transient simulations, and have not yet been examined by experimental or field measurements, leaving a gap before practical engineering deployment.

Besides the data-demand challenge, deep learning models generally suffer from insufficient interpretability in practical applications: their diagnostic decisions lack a transparent reasoning chain, which to some extent limits field maintenance personnel’s trust in and adoption of the diagnostic results. In the field of rotating machinery fault diagnosis, K. You et al. proposed a multimodal heterogeneous-fusion spiking neural network that quantifies the contribution of time–frequency features through time–frequency attention to explain the diagnostic results [62]; they further visualized and analyzed the model’s feature-learning process via role weights of a dynamic attention mechanism in sound–vibration fusion diagnosis of rolling bearings [63]. Comparable interpretability studies in the cable fault diagnosis field remain scarce at present, and these attention-visualization and feature contribution analysis approaches may appropriately inspire interpretability research in cable fault diagnosis.

## 4. Intelligent Cable Operation and Maintenance Technology Based on Digital Twin

**Figure 7:**
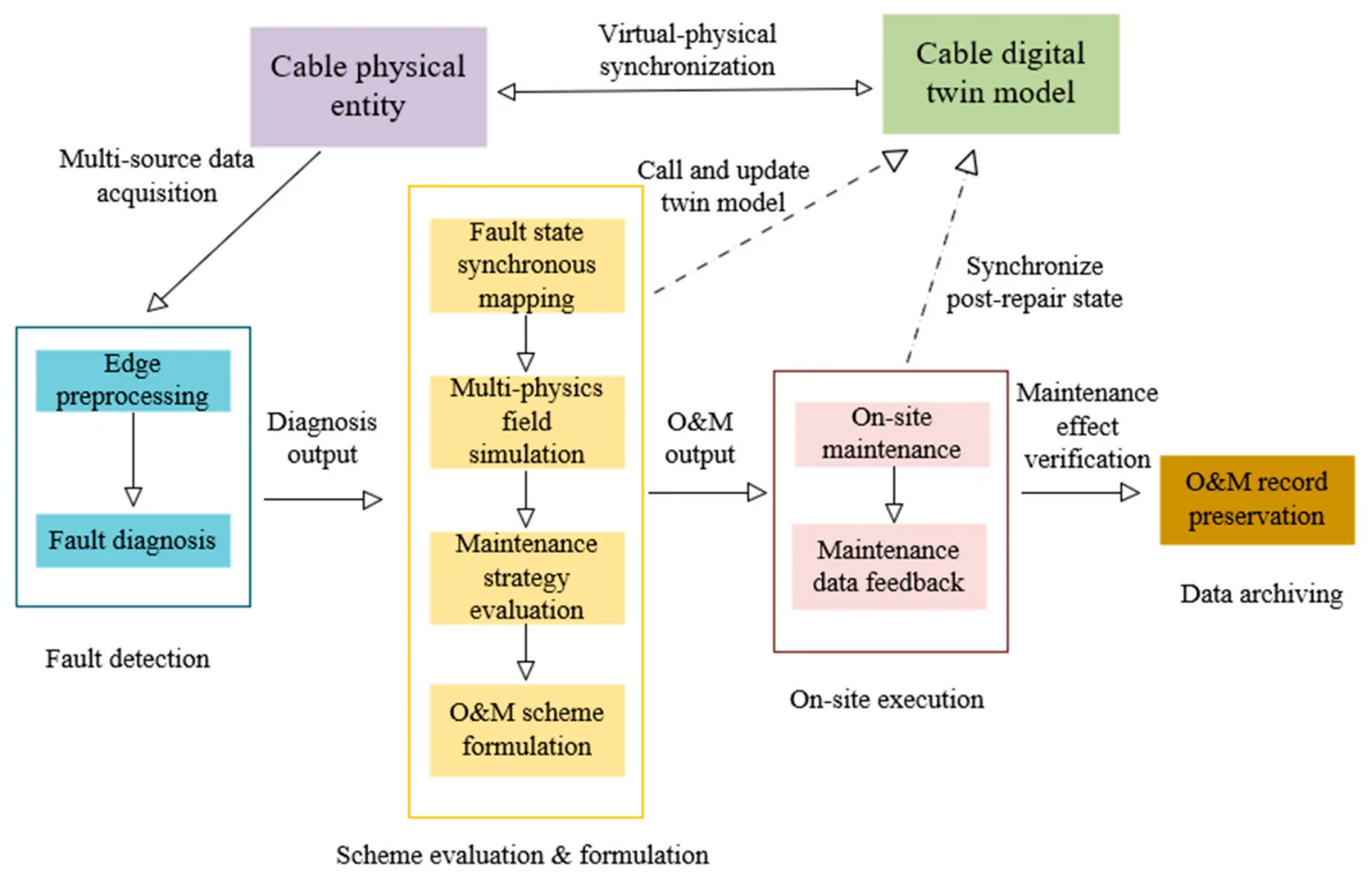
Flowchart of the intelligent O&M.

Conventional cable operation-and-maintenance modes, which rely on on-site specifications and engineering experience, have long been adopted in practical engineering. Nevertheless, with the continuous expansion of urban cable deployment scale and increasingly complex cable operating conditions, such modes gradually reveal practical shortcomings, including strong subjectivity in decision-making, low execution efficiency, and lagging verification of maintenance outcomes. With the development of digital-twin computing and artificial intelligence-related technologies, intelligent cable O&M modes have been gradually developed. They can complement and extend the existing O&M system. The complete workflow is shown in Figure 7. Multi-source data collected from the physical cable entity is subjected to edge preprocessing and fault diagnosis to produce diagnostic results. These results support fault mapping, multi-physics simulation, and maintenance-scheme formulation. The generated O&M instructions guide on-site operations, followed by data feedback. The cable digital-twin model is updated according to the equipment state after maintenance. Maintenance records are archived after effectiveness verification. Throughout the whole workflow, the physical cable entity interacts with its digital-twin model to maintain real-time virtual–physical synchronization.

### 4.1. Digital Twin Technology

**Figure 8:**
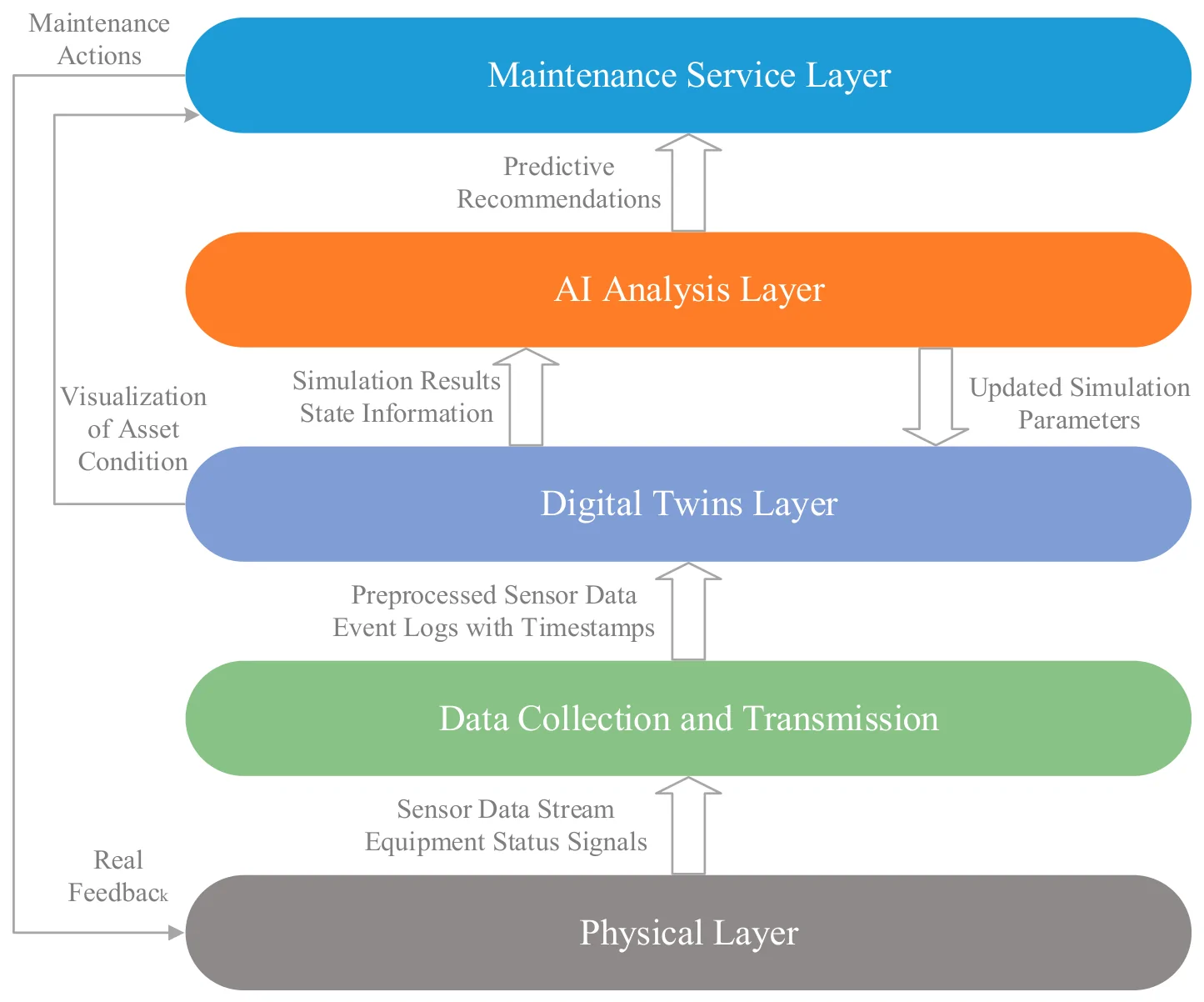
Maintenance framework based on the collaboration between artificial intelligence and digital twin.

Digital twin leverages full-lifecycle monitoring data of physical equipment to construct digital virtual models with targeted fidelity. The models can reflect the primary operating features needed for daily operation and maintenance analysis through bidirectional real-time data linkage with physical cables [64]. Digital twin systems establish mutual mapping and interactive channels linking physical cable bodies and their virtual counterparts. Through continuous data iteration and model updating, such systems aim to reproduce the primary operating behaviors of physical entities over their life cycles and support dynamic prediction. The attainable fidelity depends on model accuracy, sensor configuration, data synchronization, and validation procedures. Digital twin modules paired with artificial intelligence help diversify analytical thinking and generate more referential information to support decision-making procedures. The typical collaboration mode between AI and digital twin is illustrated in Figure 8.

As a technology that deeply integrates the Internet of Things, big data, and artificial intelligence, digital twin has received growing research attention across various fields in recent years. T. Kreuzer et al. systematically reviewed 149 AI-integrated digital twin studies covering multiple application domains such as manufacturing, healthcare, and transportation, demonstrating the broad applicability of this technology [65]. M. H. Zafar et al. positioned digital twin as a key enabling technology of Industry 5.0, examined its synergy with collaborative robots, augmented reality, and artificial intelligence, and reviewed the stages of digital-twin-based human–machine collaboration from modeling and simulation to deployment, monitoring, and optimization [66]. Digital twin technology has also been explored for power cables. For 220 kV cable lines, J. Wang et al. developed a digital twin operation and maintenance platform and applied it to a cable line of the State Grid. The platform integrates multi-sensor perception, BIM modeling, edge computing, and remote control to build a comprehensive virtual mapping system. LoRa communication is employed for low-power long-distance data transmission, and front-end edge devices handle local data processing. The system provides early warning and linkage control against tunnel external damage, insulation partial discharge, channel settlement, and abnormal environmental parameters, and supports the formulation of maintenance strategies based on status analysis [67]. Overall, the above studies show prospects of digital twin in cable O&M, but its application is still in its infancy. Most systems focus on architecture construction and functional demonstration, and quantitative assessment of fidelity and O&M benefits remains to be strengthened. Meanwhile, most relevant discussions remain at the conceptual and framework level, and exploration oriented toward practical deployment and operational verification is relatively limited.

### 4.2. Optimization of Operation and Maintenance Schemes Based on Simulation

The fault diagnosis module clarifies core information including defect types, defect locations, and impact levels. On this basis, digital twin technology constructs a high-fidelity dynamic virtual mapping model for physical cable entities. This model integrates cable structure parameters, full life-cycle operation and maintenance data, real-time environmental data, and equipment operation baseline data. Thereby, fault scenarios and current equipment states are reproduced in virtual space, and dynamic data interaction channels are built between physical entities and virtual models.

Multi-physics simulation recreates typical cable fault conditions using collected field monitoring data and builds virtual–physical data channels. When analyzing maintenance schemes quantitatively, the obtained results fit well with scenarios covered by existing monitoring records. The operation of cables is affected by the coupling of multiple physical fields such as electric field, temperature field, stress field, and environmental field. For complex faults involving multiple physical processes, single-parameter monitoring may not fully capture the fault evolution mechanism. Multi-physics simulation can model the coupling effects of multiple fields and provide a quantitative basis for scheme comparison and selection, which supports the quantitative comparison of maintenance schemes. L. Harbour et al. pointed out that current multi-physics simulation technology is developing towards high fidelity and large-scale parallel computing. Simulation frameworks represented by MOOSE are equipped with diversified discrete methods, high-performance solvers, and GPU acceleration capabilities, and are applicable to complex multi-field coupling working conditions and align with technical specifications for integrated digital twin implementations [68]. However, high-fidelity simulation is computationally intensive. It is limited when relying solely on hardware acceleration in the context of digital twins, where the demand for real-time performance is increasingly critical. To address this challenge, model order reduction techniques reduce the computational scale through low-dimensional projection, while surrogate models approximate the original simulations by learning the input-output mapping from simulation data. Both approaches can reduce computation time, offering a way to balance simulation accuracy with real-time responsiveness.

Multiple candidate operation and maintenance schemes are formulated according to fault diagnosis results with the assistance of multi-physics simulation technology and the multi-scenario simulation deduction capability of the digital twin. Various schemes usually restrict one another in dimensions including safety risk, implementation efficiency, resource consumption, and defect elimination effect, so optimal decision-making cannot be achieved by a single indicator. The multi-task multi-objective evolutionary algorithm (MTMOEA) proposed by T. Liu et al. can be used to address such problems. The algorithm integrates dynamic constraint updating and knowledge transfer strategies and constructs a collaborative optimization framework for main tasks and dual auxiliary tasks. It can balance multiple conflicting objectives under complex constraint conditions [69]. The introduction of this algorithm enables the screening of candidate maintenance schemes with balanced performance across multiple objectives.

### 4.3. Decision Formulation and Field Execution

Operation and maintenance plans need to comprehensively consider multi-dimensional information, including equipment performance baselines, resource inventory data, and on-site condition constraints, in the transformation to on-site execution decisions. Traditional empirical judgment fails to achieve rapid and accurate decision matching. Q. Pan et al. constructed the PSO-XGBoost model, which can extract hidden rules from massive data and realize automatic parameter optimization through the particle swarm optimization algorithm. This algorithm alleviates the limitation of experience-dependent manual parameter tuning. In the analysis of multi-source cable operating data, its key performance indicators exceed 98%, outperforming traditional methods such as SVM and random forest in the reported case [70]. Such algorithms can be used for spare parts allocation, personnel division, and operation specification formulation in on-site work, facilitating decision execution.

To ensure the consistency between decision-making and on-site execution, a bidirectional physical-virtual interaction architecture is built based on Internet of Things technology to realize real-time data synchronization throughout the operation and maintenance process. However, the operation and maintenance site faces challenges such as massive data volume, high real-time requirements, and large cloud transmission delay. X. Gu et al. applied edge computing to power cable monitoring. By sinking data analysis and preprocessing capabilities to network edge nodes, they reduced transmission delay and improved the local response of real-time on-site monitoring and data processing. The actual improvement varies with network architecture and workload conditions [71]. Edge computing is adopted to complete preprocessing and real-time discrimination of sensor data locally, and valid information is further synchronized to the virtual model, so that data synchronization efficiency and response speed can be improved. Operators are able to visually follow maintenance progress and carry out real-time inspections for operational standardization when applying this mode.

### 4.4. Effect Verification and Data Archiving

Real-time data collected on-site allows the simulation verification of the digital twin model to evaluate O&M performance. The specific method is to restore actual load and environmental conditions in the virtual model and compare core indicators before and after operation and maintenance, such as insulation performance, current-carrying capacity, and partial discharge signal strength. Within the scope of visible fault disposal, this workflow helps judge the completion degree of defect treatment and provides a basis to check if maintenance items match preset evaluation standards.

After effect verification, all operation and maintenance data are systematically archived following the full life cycle data management concept. These data are synchronously updated to the digital twin model and operation and maintenance database [72]. The real-time iteration of the virtual model maintains correspondence with the status of physical cables. A full life-cycle operation and maintenance archive of equipment is established. Records stored in the archive can be consulted when dealing with faults of the same type. Analyzing these accumulated data via big data means helps sum up regular tendencies reflected in historical operation and maintenance cases. These historical materials can be incorporated into the intelligent closed-loop O&M system and used as one of the resources to standardize and digitize daily maintenance work.

## 5. Challenges and Future Prospects

### 5.1. Existing Challenges

Artificial intelligence is gradually being integrated into the operation and maintenance of power cables, providing new approaches to overcome the limitations of conventional maintenance modes. However, power cables are characterized by underground concealed laying, long-distance chained transmission, and hidden insulation degradation processes, and the application and deployment of artificial intelligence technology in cable operation and maintenance still face several technical constraints.

High-quality field fault samples are scarce, and the performance of laboratory-simulated models is limited when transferred to on-site cable operating conditions. Unlike power equipment such as transformers that can be de-energized for sampling, urban power cables are mostly laid in enclosed underground environments such as tunnels and cable trenches, where various insulation defects and joint hazards are relatively concealed and routine inspections often cannot fully cover them. The overall failure rate of cables is low, with scattered fault types, and the high-quality labeled samples available for model training are limited; moreover, the sample sizes across different types vary greatly, further causing class imbalance in datasets. Restricted by management systems and inconsistent data formats, the sharing of monitoring data among operation and maintenance organizations is difficult, and scattered fault records can hardly be aggregated into a sufficiently large sample pool. In contrast, conventional physics-based diagnostic methods rely on insulation mechanisms and traveling wave theories and have lower requirements for labeled samples, but their several idealized simplifications have limited adaptability to complex on-site conditions. Although data-driven artificial intelligence models possess strong feature-fitting capabilities, their performance largely depends on sufficient labeled samples consistent with real operating conditions; laboratory and simulation datasets often simplify on-site electromagnetic interference, joint losses, and disturbances from laying environments, resulting in distribution deviations from real underground-cable signals, and the identification performance achieved in laboratory tests has not yet been fully verified by field measurements.

Dynamic variations in grid operating conditions limit the robustness of models under complex field conditions. The insulation-related electrical properties of urban XLPE cables are affected by operating temperature and load status: the diurnal and seasonal fluctuations of distribution-network loads dynamically change the conductor temperature, thereby altering the electric-field distribution and the activation conditions of insulation defects, so that tiny defects dormant under low-load conditions may be activated during peak-load and high-temperature periods, resulting in non-stationary drift of fault features. Meanwhile, monitoring signals suffer amplitude attenuation during long-distance transmission. Impedance discontinuity at intermediate joints and cross-bonded grounding configurations further cause waveform distortion, which, superimposed with on-site electromagnetic noise, leads to some degradation in monitoring data quality. However, most existing AI models are still trained on datasets obtained under steady-state operating conditions, and their adaptability to such temperature fluctuations, signal distortion, and noise interference is limited. Some models experience performance degradation under distribution shift, and stably extracting valid fault features under diverse working conditions remains difficult.

Limited decision-making reliability and insufficient interpretability constitute another challenge. Unlike conventional diagnostic methods such as traveling wave location and insulation resistance testing that rely on clear physical principles, fault judgments given by AI models often output only categories or probabilities without traceable physical evidence. Once a cable fault is misjudged, it often involves the outage scheduling and on-site maintenance plans of multiple lines; in scenarios of live cable testing or emergency disposal, operation and maintenance personnel can hardly decide whether to de-energize solely based on the judgment of a black-box model, and tend to conduct manual review or temporarily refrain from adopting the result, so that AI outputs can hardly be directly translated into maintenance actions. Meanwhile, because the internal decision-making process of the model is opaque, once a misdiagnosis occurs, it is difficult to determine from a physical perspective whether the problem lies in data quality, model bias, or the fault itself, which also hinders the targeted improvement and continuous iteration of cable diagnostic systems.

### 5.2. Future Prospects

Addressing the above challenges, further research can be promoted from several aspects.

To address the obstacle of insufficient field fault samples, efforts can be made in both data acquisition and sample expansion. In terms of data acquisition, unified fault-data collection and labeling standards, covering fault types, sampling rates, waveform settings, and labeling criteria, should be promoted to aggregate scattered records into a reusable dataset. In terms of sample expansion, data augmentation, semi-supervised learning, and physics-simulated synthetic samples have been preliminarily explored in cable fault diagnosis. Further developing multi-condition physical simulation to reproduce on-site noise and joint-induced waveform distortion, while validating the synthetic-to-real gap on field-measured waveforms, can be a near-term focus. Although federated learning, which enables multiple parties to collaboratively train models without sharing raw data, has shown the potential to alleviate data silos and protect privacy in rotating machinery and industrial equipment fault diagnosis, its application in the field of cable operation and maintenance is still relatively rare, and its actual effectiveness in cross-organization modeling remains to be verified in the field.

Regarding operating-condition adaptability, the effects of load and temperature on cable insulation degradation, ampacity, and life have been extensively modeled. Yet introducing such operating-condition coupling into AI fault diagnosis and enabling models to adapt to variations in load and temperature remains in an early stage. A concrete direction is to construct load–temperature coupled field datasets and evaluate domain-adaptive transfer learning on 110 kV lines across different seasons, with the aim of maintaining recognition accuracy when operating conditions shift. In the traveling wave direction, AI fault location based on wave velocity and arrival time has been validated through simulation or laboratory experiments on cables and hybrid lines. Even so, regarding the waveform distortion caused by long-distance attenuation and joint impedance discontinuity, embedding traveling wave mechanisms into pre-processing to correct distortion still lacks dedicated validation in AI cable diagnosis. Field-measured traveling wave waveforms could serve as benchmark data for such validation.

In terms of interpretability, general explainable frameworks such as SHAP and LIME have been used in some power-equipment fault diagnosis cases to quantify feature contributions and construct local surrogate models. Nevertheless, this experience mainly comes from broader power-equipment diagnosis, and the feature weights they produce mostly reflect data-driven statistical correlations rather than the intrinsic mechanisms of degradation processes such as water trees and electrical trees in XLPE insulation, so direct transfer to cable fault diagnosis remains limited. Physics-informed learning embeds physical mechanisms such as telegrapher-equation residuals, traveling wave propagation laws, and electro-thermal conduction equations as loss terms into networks, and has recently shown in cable research the effects of improving small-sample accuracy and cross-condition robustness. A possible research direction is to embed physics-based models of electrical-tree growth into the loss function and interpret PRPD/PRPS spectral patterns under such physical constraints, linking discharge-pattern evolution to tree growth stages, with the results evaluated against field failure cases. Constraining the model solution space with physical laws is expected to improve the correspondence between diagnostic results and fault evolution mechanisms, compared with purely data-driven approaches. Overall, mechanism-level interpretable diagnosis remains an emerging direction in cable fault diagnosis, and establishing the correspondence between fault features and insulation defects through the above routes is a near-term goal.

## 6. Conclusions

The application status of artificial intelligence in the three key processes of cable operation and maintenance oriented towards fault disposal is systematically reviewed. These processes include condition monitoring, fault diagnosis, and intelligent operation and maintenance. The applicable boundaries and deployment constraints of various technologies across different cable O&M scenarios are examined. These efforts are conducted through analysis of monitoring technologies, AI diagnostic models, and digital twin collaboration.

From a maturity perspective, the perception layer monitors key physical quantities such as partial discharge, sheath circulating current, and temperature through sensing methods including HFCT, UHF, and acoustic emission. These technologies have been applied in engineering practice. Multi-sensor complementary configurations have been adopted in multiple studies to improve defect detection reliability. Communication and distributed optical fiber sensing technologies such as LoRa, NB-IoT, and DAS have been deployed in several cable monitoring systems. However, the intelligent architecture that organizes these perception and transmission nodes in layers such as front-end preprocessing, gateway aggregation, and cloud analysis remains an emerging stage. The diagnosis layer, including CNN, LSTM, and Transformer methods, has been relatively well validated under simulation and laboratory conditions. Field validation based on measured data remains limited. Data augmentation and physics-informed learning have been initially explored in cable fault diagnosis. The decision layer involves O&M workflows that combine digital twins with AI. Pilot platforms have emerged on cable lines. However, multi-physics simulation, multi-objective optimization, and data mining decision support are still mainly in the method exploration stage. Effectiveness validation for cable O&M remains relatively limited. Overall, current research has made progress mainly under laboratory conditions. Yet, moving from laboratory validation to large-scale field deployment of cable O&M requires continued improvement in data foundation, operating-condition adaptability, and decision credibility.

For researchers, future attention can be directed toward high-quality acquisition and labeling standards for field fault samples, design of operating-condition adaptive models, and physics-constrained interpretable diagnosis. For engineering practice, corresponding data quality assurance mechanisms can be established in areas such as front-end measurement anti-interference, edge preprocessing, and cloud-diagnosis collaboration. These efforts will gradually drive AI technology from laboratory validation toward large-scale deployment in cable O&M field applications.

## Figures and Tables

**Table 1:** Comparison of various partial discharge measurement technologies.

Method	Measured Signal	Frequency Bandwidth/MHz	Advantages	Disadvantages
Ultra-High Frequency (UHF) Method	Electromagnetic wave	300–3000	High anti-interference performance and high signal-to-noise ratio (SNR).	Rapid attenuation of UHF signals.
High-Frequency Current Transformer (HFCT) Method	Pulse current	0.1–100	High-sensitivity PD apparent charge measurement, good applicability.	Band overlaps with on-site electromagnetic interference, resulting in low SNR.
Coupling Capacitor (CC) Method	Pulse current	0.1–100		
Acoustic Emission (AE) Method	Acoustic wave	0.0001–0.3	Immunity to electrical signal interference.	Severe acoustic attenuation and low detection sensitivity.

**Table 2:** Comparison of CNN-based models for power cable fault diagnosis.

Authors	Architecture	Task	Data & Performance	Deployment
Tu et al.	CNN + SE attention + LSTM	High-voltage cable sheath cross-bonding error detection	36,000 simulation samples; acc >98%	Simulation-only; no field data
Wang et al.	DWT + Lorenz chaos + CNN	XLPE cable partial discharge insulation diagnosis (four defect types)	760 lab samples; acc 97.5%	Offline lab only
Lan et al.	CNN (2D time–frequency input)	HV cable online fault localization via sheath traveling wave signals	Data-efficient; simulation; high accuracy	Low-cost sensors; simulation-only; performance drops under heavy noise
Said et al.	FrDCT + SVD + BSVM + 1D-CNN	Medium-voltage cable fault classification and location	ATP/EMTP simulation; acc 99.6%; loc error 0.095%	Real-time capable; simulation-only
Zhao et al.	CNN-Transformer hybrid	Cable insulation defect classification based on ultrasonic signals	Measured signals; acc 100%	Non-invasive, low-cost
Chen et al.	ARDDMA-Net (diffusion denoising + ResNet-1D)	Cable partial discharge identification under low-SNR conditions	3200 lab samples; acc 98.36%; noise-robust	Lightweight; lab-verified only

**Table 3:** Performance comparison of different deep learning models in cable fault diagnosis.

Model	Computational Complexity	Applicable Fault Types	Data Requirement	Real-Time Processing Capability
CNN	Grows linearly with kernel size and number of channels	Excels at waveform local feature extraction & PRPD spectrum recognition; sensitive to transient fault features	Medium; stable performance via data augmentation/pre-training for small samples	Highly parallel convolution with low inference latency, suitable for edge computing & online monitoring
LSTM	Grows linearly with sequence length and hidden dimensions, higher than CNN	Excels at long-range dependency modeling of time series	Relatively high; complete time series required, poor performance with limited samples	Serial computation restricts parallel capacity, leading to relatively high inference latency
Transformer	Grows quadratically with sequence length, highest among the three	Specialized in global dependency modeling for complex faults with cross-time-step correlations	Very high; prone to overfitting/underfitting on small datasets	Heavy self-attention computation yields maximum latency, more applicable to offline analysis
CNN+LSTM	Higher than single model	Applicable to fault classification and localization	Moderately high; transfer learning delivers comparable performance with limited data	Between CNN and LSTM
CNN+ Transformer	Higher than single model	Applicable to defect detection with tiny targets, occlusion and complex backgrounds	Very high; inherits Transformer’s high data dependency	Between CNN and Transformer

## Data Availability

No new data were created or analyzed in this review.
